# Supplementary material for: Risk for short-term undesirable outcomes in older emergency department users: Results of the ER2 observational cohort study
Source: PLoS One. 2021 Aug 11;16(8):e0249882. doi: 10.1371/journal.pone.0249882 (PMC8357090; doi:10.1371/journal.pone.0249882)
Supplement: Attachment — Submitted filename: Responses_Comments_PONE-D-20-15983.docx [file pone.0249882.s001.docx]

Manuscript:

- ID#: **PONE-D-20-15983**
- MS TITLE: **"Risk for short-term undesirable outcomes in older emergency department users: Results of the ER2 observational cohort study"**
- Submitted for publication in **PLOS ONE**

We thank the Editor and the Reviewer for their thoughtful review. As suggested, we performed a revision of our manuscript. We think that, after following the Reviewer’s comments, the revised version is a significantly improved manuscript. Please, find below our point-by-point responses to the comments and queries raised by the Editor and the Reviewer.

**Comment of the Editor**

**Comment 1:** *“Please ensure that your manuscript meets PLOS ONE's style requirements, including those for file naming.”*

We confirm that our manuscript meets PLOS ONE's style requirements.

**Comment 2:** *“We note that you have provided funding information that is not currently declared in your Funding Statement. However, funding information should not appear in the Acknowledgments section or other areas of your manuscript. We will only publish funding information present in the Funding Statement section of the online submission form. Please remove any funding-related text from the manuscript and let us know how you would like to update your Funding Statement. Currently, your Funding Statement reads as follows: "No, the funders had no role in study design, data collection and analysis, decision to publish, or preparation of the manuscript.”*

We removed the funding Information section from the manuscript as requested. Please see correction on page 22.

**Comment 3:** *“We note that you have indicated that data from this study are available upon request. PLOS only allows data to be available upon request if there are legal or ethical restrictions on sharing data publicly. For information on unacceptable data access restrictions, please see…”*

We thank for this information. Effectively, there is an ethic restriction from the Jewish General Hospital (Montreal, Quebec, Canada) which approved the study to access to the data. So, Data and materials are available on request. Request should be sent to the corresponding author: Olivier Beauchet, MD, PhD; Department of Medicine, Division of Geriatric Medicine, Sir Mortimer B. Davis - Jewish General Hospital, McGill University, 3755 chemin de la Côte-Sainte-Catherine, Montréal, QC H3T 1E2, Canada; E-mail: [olivier.beauchet@mcgill.ca](mailto:olivier.beauchet@mcgill.ca). All requests needs a cover letter explaining the objective, justification and the referent ethic committee.

**Comment 4:** *“In your revised cover letter, please address the following prompts: a) If there are ethical or legal restrictions on sharing a de-identified data set, please explain them in detail (e.g., data contain potentially identifying or sensitive patient information) and who has imposed them (e.g., an ethics committee). Please also provide contact information for a data access committee, ethics committee, or other institutional body to which data requests may be sent.”*

We added the requested information in our revised cover letter as suggested. Please see revised cover letter.

**Comment 5:** *“In your revised cover letter, please address the following prompts: If there are no restrictions, please upload the minimal anonymized data set necessary to replicate your study findings as either Supporting Information files or to a stable, public repository and provide us with the relevant URLs, DOIs, or accession numbers. Please see* [*http://www.bmj.com/content/340/bmj.c181.long*](http://www.bmj.com/content/340/bmj.c181.long) *for guidelines on how to de-identify and prepare clinical data for publication. For a list of acceptable repositories, please see* [*http://journals.plos.org/plosone/s/data-availability#loc-recommended-repositories*](http://journals.plos.org/plosone/s/data-availability#loc-recommended-repositories)*. We will update your Data Availability statement on your behalf to reflect the information you provide.”*

We provided requested information in the revised cover letter as suggested. Please see revised cover letter.

**Comment 6:** *“PLOS requires an ORCID iD for the corresponding author in Editorial Manager on papers submitted after December 6th, 2016. Please ensure that you have an ORCID iD and that it is validated in Editorial Manager. To do this, go to ‘Update my Information’ (in the upper left-hand corner of the main menu), and click on the Fetch/Validate link next to the ORCID field. This will take you to the ORCID site and allow you to create a new iD or authenticate a pre-existing iD in Editorial Manager. Please see the following video for instructions on linking an ORCID iD to your Editorial Manager account:* [*https://www.youtube.com/watch?v=_xcclfuvtxQ*](https://www.youtube.com/watch?v=_xcclfuvtxQ)*.”*

The corresponding author (Cyrille P Launay) has a *an ORCID ID.* Please see correction on online system.

**Comment 7:** *“Please amend the manuscript submission data (via Edit Submission) to include authors Kevin Galery, Christine Vilcocq,  Marc Afilalo, Olivier Beauchet.”*

We made the correction as suggested by the Editor.

**Comment of the reviewer #1**

**Comment 8:** *“1. Is the manuscript technically sound, and do the data support the conclusions?
The manuscript must describe a technically sound piece of scientific research with data that supports the conclusions. Experiments must have been conducted rigorously, with appropriate controls, replication, and sample sizes. The conclusions must be drawn appropriately based on the data presented. Reviewer #1: Partly.”*

We thank the reviewer for its review and made requested changes. Please see corrections through the revised manuscript and please see also answers to the Reviewer’s Comments below.

**Comment 9:** *“2. Has the statistical analysis been performed appropriately and rigorously? Reviewer #1: I Don't Know.”*

This answer does not require an answer form author.

**Comment 10:** *“3. Have the authors made all data underlying the findings in their manuscript fully available? The* [*PLOS Data policy*](http://www.plosone.org/static/policies.action#sharing) *requires authors to make all data underlying the findings described in their manuscript fully available without restriction, with rare exception (please refer to the Data Availability Statement in the manuscript PDF file). The data should be provided as part of the manuscript or its supporting information, or deposited to a public repository. For example, in addition to summary statistics, the data points behind means, medians and variance measures should be available. If there are restrictions on publicly sharing data—e.g. participant privacy or use of data from a third party—those must be specified. Reviewer #1: No.”*

We made corrections as suggested by the Reviewer’s Comment below. Please see corrections through the revised manuscript and please see also answers to the Reviewer’s Comments below.

**Comment 11:** *“4. Is the manuscript presented in an intelligible fashion and written in standard English? PLOS ONE does not copyedit accepted manuscripts, so the language in submitted articles must be clear, correct, and unambiguous. Any typographical or grammatical errors should be corrected at revision, so please note any specific errors here. Reviewer #1: No.”*

A revision of the manuscript has been made by a native English physician. Please see corrections through the revised manuscript and please see also answer to the Reviewer’s Comment 24

**Comment 12:** *“5. Review Comments to the Author. Reviewer #1: This manuscript is meant to confirm the ability of the ER2 to predict hospitalization and length of stay in the ED and hospital in older adults admitted to the emergency department on a stretcher. This has been established in prior studies but not in Canada. It also focuses on maintenance of completion rates and time to completion as proxies for usability.”*

We thank the reviewer for this good summary of our manuscript.

**Comment 13:** *“Review Comments to the Author. Reviewer #1: I believe the manuscript has potential, even though it's primarily confirmatory…”*

We thank the Reviewer for this positive comment on our manuscript.

**Comment 14:** *“Review Comments to the Author. Reviewer #1: … but it is currently limited by apparent inconsistencies in methods and presentation and somewhat awkward writing in places that makes it difficult to follow.”*

We followed the recommendations and suggestions formulated by the reviewer with the objective to improve the Methods and the presentation of results. Please see corrections through the manuscript and also answer to the comments below.

**Comment 15:** *“Review Comments to the Author. Reviewer #1: My biggest concern is the apparent inconsistency between the methods and presentation of results, which causes confusion.*

The reviewer is right, and we made corrections with the objective to be more consistent between Objectives, Methods and the Presentation of results. First, we propose to delete the Table 1 which shows the comparisons of baseline characteristics of participants on stretcher visiting Emergency Department with Emergency Room Evaluation and Recommendation (ER^2^) assessment filled and not filled. However, we propose to explain the main results of in the Section Methods in the subsection 2.1 Study design and population and to show the Table in supplementary data. This Table provides information not directly related to the objectives of the study but its information may be helpful for the readers to understand the differences between older ED users who had and who had not ER^2^ assessment, this information being an indirect information of feasibility of ER^2^ assessment in the older ED users. Please see correction from page 5 to 17.

**Comment 16:** *“Review Comments to the Author. Reviewer #1: I am also concerned about the apparent lack of reference group in regression models comparing outcomes among categories, which doesn't make sense.”*

We thank the reviewer for this comment, and we apologize for the lack of information provided in the first version of our manuscript which lead to issue for understanding both linear and Cox regression models. In fact, in both cases we performed two categories of models:

- The first model used as the reference groups the two other groups of ER^2^ risk-level merged together

The second model as shown in the Table used the ER^2^ low risk-level as the referent group.

We added more information in the Methods and Results Section and in the Table 1. Please see corrections on pages 9,16 and 17.

**Comment 17:** “*Review Comments to the Author. Reviewer #1: Major concerns: I’m confused by table 3 because every risk category has a parameter estimate. I would expect one of these to be the reference group against which others are compared. What is the reference group?”*

The reviewer is right. Table 3 was difficult to understand because of lack of information. We performed two categories of models:

- The first model used as the reference groups the two other groups of ER^2^ risk-level merged together
- The second model as shown in the Table used the ER^2^ low risk-level as the referent group.

Please see corrections on pages 9,16 and 17.

**Comment 18:** *“Review Comments to the Author. Reviewer #1: Major concerns: There are inconsistencies in the description of the statistical analysis and presentation of results. The statistical methods seem to indicate that both linear regression and Cox regression were used to study time to discharge from the ED and hospital. The description of the Cox model suggests there were two models, but table 3 suggests a Cox model was used to look at hospital admission as an outcome.”*

We thank the reviewer for this comment. In the first version of our manuscript, we made the choice to present both linear and Cox regression in the same Table. The sentence in Statistical analysis Section and the tile were confusing. In fact, the regression models show the association of the length of stay in emergency department and hospital (linear regression) as well as hospital admission (Cox regression) used as dependent variables with separated model for each outcome with Emergency Room Evaluation and Recommendation risk-levels used as independent adjusted by participants’ baseline characteristics in older Emergency Department users on stretcher (n=1,800). We made change to clarify the purpose of these analyses. Please see correction on pages 9 and 16.

**Comment 19:** *“Review Comments to the Author. Reviewer #1: Major concerns: Figure 3 is described as time to discharge from the ED, while the statistical analysis section describes a Kaplan-Meier curve of time to discharge from hospital. Is this time to discharge from ED or hospital to a non-hospital location? I’m guessing so since this seems like the best strategy, but it’s not consistent with the presentation of the Cox model which seems like the related analysis or with how it’s described. How was death in the hospital or ED handled in this analysis? Were patients censored if they died?”*

We apologize for this error. The figure 3 showed the elapsed time to discharge from hospital to a non-hospital location by survival Kaplan-Meier curves. Effectively, patients were censored if they died. We made changes in the revised version of the manuscript. Please see corrections on pages 9 and 17.

**Comment 20:** *“Review Comments to the Author. Reviewer #1: Other issues: I believe a more coherent story could be told if the rationale for certain measurements was stated up front, e.g. the completion rates and time to completion being proxies for usability. As I read, I was confused as to the purpose of comparing these rates and times to completion by month. Even in the discussion it seems like the interpretation in relation to usability could be clearer.*

The reviewer underscored an important topic related to the outcomes used as markers of usability which refers to evaluate a product by testing it on users. More precisely, usability is a measure of how well a specific user in a specific context can use a product to achieve a defined goal efficiently and satisfactorily. A tool usability depends on how well its features accommodate users’ needs and contexts. This context in our case is a busy Emergency Department (ED)and a short period of time to make decision and discharge the patient from ED. Thus, a clinical tool usable in daily practice of ED should be at least:

- Ease to learn (*i.e.,* new users can accomplish goals related to the tool easily and even more easily when repeated). In our case, we propose to assess this component of usability with time the time to fill and record ER^2^ items in the patient’s digital file and its evolution with time over the first months of its implementation in the ED daily practice.
- Efficient (*i.e.,* users can perform the tasks related to the tool quickly through the easiest process) effectiveness (*i.e.,* it supports users in completing actions accurately. To estimate this component, we decided to use a simple surrogate measure which was the evolution of the *ratio* of ER^2^ assessment completed (*i.e.,* number of older ED users with an ER^2^ score / number of older ED users without an ER^2^ score) over the first months of its implementation in the ED daily practice. The hypothesis was that a high ratio and its increase with time were indirect markers of efficiency and effectiveness.

We added more information in the revised version of our manuscript. Please see corrections on pages 4 and 5.

**Comment 21:** *Also, the purpose of table 1 is never really clearly described. Late in the discussion section it's mentioned that the population may not be representative, without referring back to this table. Describing the purpose of various measures, creating this table, etc., would tell a clearer story.”*

We agree with the reviewer. This lack of information on the propose of the Table leads to confusion. Because information on Table 1 are not directly related to the objective of the study, we decided to provided information on Table 1 in supplementary data section. Please also answer to the Comment 15 and corrections from page 5 to 17.

**Comment 22:** *“Review Comments to the Author. Reviewer #1: Other issues: I’m not sure I agree with calling a long LOS in the ED and hospital or hospital admission “short-term ED adverse events.” They are certainly outcomes of more severe conditions, but “adverse event” usually specifically refers to harm from medical care. I think something more generic like “worse outcomes” would be better.”*

We followed the reviewer suggestion and change “adverse outcomes” by “undesirable outcomes”. Please corrections of terminology through the revised manuscript.

**Comment 23:** *“Review Comments to the Author. Reviewer #1: Other issues: In the results section of the abstract, I’m not sure why the betas for the high-risk group are defined as >/=. Aren’t they just point estimates, such that this should be” =”? Also, presenting these as parameter estimates is not clear without specifying the medium risk group as the reference, but I remain confused about what these are being compared to since every group has a parameter estimate.”*

We agree with the Reviewer and we made the requested changes. Please see also answer to the Comment 17. Corrections has been made on page 3 in the Methods Abstract subsection.

**Comment 24:** *“Review Comments to the Author. Reviewer #1: Other issues: The clarity of writing could be improved in a number of places, e.g. on page 6 when describing high risk. The sentence starting with “An association of the two major items…” could simply state that one major item and at least one other major or minor item defines the high-risk level.”*

We revised the manuscript and tried to simplify it as suggested by the Reviewer. Please corrections through the revised manuscript.

**Comment 25:** *“Review Comments to the Author. Reviewer #1: Other issues: Why are the rates of ER2 completion and hospitalization described as ratios instead of just percents? Ratios seem like an unnecessarily complicated way of presenting the data. Figure 1 actually shows percents, so I’m not sure why the text and title suggest that the ratios are being presented.”*

We followed the reviewer suggestion and change the terminology as requested. Please see correction on pages 5 and 8 to 11.

**Comment 26:** *“Review Comments to the Author. Reviewer #1: Other issues: Why did you choose to adjust for Canadian emergency department triage and acuity scale level in models? Were you looking for value added beyond this severity measure? That’s probably fine, but that could be discussed for context since both are getting at acute health status to some degree.”*

We thank the Reviewer to highlight this questioning. ER^2^ does not take into consideration the reasons of ED visits and its severity which is usually an acute disease influencing directly the occurrence of the short-term ED undesirable outcomes examined in our study. For instance, an acute disease may decompensate in cascade chronic morbidities cumulated in older ED users, leading to multiple acute organ decompensations and disabilities and exposing to long length of stays and hospitalisations. As suggested by the reviewer we made correction in the revised discussion. Please see corrections on page 21.

**Comment 27:** *“Review Comments to the Author. Reviewer #1: Other issues: Was the analysis of LOS in the ED restricted to patients who were not hospitalized? It seems not. This seems like a good idea since very sick patients might move quickly to the hospital.”*

We did not limit the analysis of LOS in the ED to a subgroup of patients. All pateints (n=1800) were included in the analysis.

**Comment 28:** *“Review Comments to the Author. Reviewer #1: Other issues: I believe “residences” is used to describe nursing homes or some kind of residential care. I think “residences” means wherever someone lives to most people, so different language for this category would be helpful.”*

We thank the Reviewer to underscore this terminology issue. We agree with Reviewer when she/he propose to use a more consensual terminology like nursing home which may be defined as a facility for the residential care of elderly or disabled people. We made changes through the revised manuscript.

**Comment 29:** *“6. PLOS authors have the option to publish the peer review history of their article (*[*what does this mean?*](https://journals.plos.org/plosone/s/editorial-and-peer-review-process#loc-peer-review-history)*). If published, this will include your full peer review and any attached files. If you choose “no”, your identity will remain anonymous but your review may still be made public.* ***Do you want your identity to be public for this peer review?*** *For information about this choice, including consent withdrawal, please see our* [*Privacy Policy*](https://www.plos.org/privacy-policy)*. Reviewer #1: No.”*

This comment does not require an answer from authors.
