# Supplementary material for: Risk for short-term undesirable outcomes in older emergency department users: Results of the ER2 observational cohort study
Source: PLoS One. 2021 Aug 11;16(8):e0249882. doi: 10.1371/journal.pone.0249882 (PMC8357090; doi:10.1371/journal.pone.0249882)
Supplement: Attachment — Submitted filename: Responses_Comments_PONE-D-20-15983-R3.docx [file pone.0249882.s002.docx]

Manuscript:

- ID#: **PONE-D-20-15983**
- MS TITLE: **"Risk for short-term undesirable outcomes in older emergency department users: Results of the ER2 observational cohort study"**
- Submitted for publication in **PLOS ONE**

We thank the Reviewer for her/his second review. As suggested, we performed a revision of our manuscript. Please, find below our point-by-point responses to the comments and queries raised by the Reviewer.

**Comment of the Reviewer**

**Comment 1:** *“This paper still suffers from many of the same issues as in the last submission. The models that lumped groups are better explained, but I still don’t understand the purpose of modeling in this way. Model 2 in table 2 should be adequate and makes a lot more sense than the models described as model 1. I don’t think the model 1 models add anything and believe that they detract by being confusing.”*

The reviewer is right and we followed the reviewer’s suggestion. We removed the Model 1 in Table 2 which initial objective was to describe the results of an unadjusted statistical model. We also consider that results provided by Model 2 are more relevant. We think that presented this way the results are clearer and the subject more fluent. Please see corrections on pages 11 and 19.

**Comment 2:** *“There are other internal inconsistencies in the writing and presentation. Please make sure all p-values and comparisons are accurately described in the text, and are consistent with the tables and figures.”*

The reviewer is correct and we apologize for those mistakes. We made the requested changes. Please see corrections on pages 14, 15 and 20.

**Comment 3:** *“Page 5 and 9 describe the ratio of completed to not completed assessments as the percent, but this is not the percent. The denominator for a percent should be number of older adult ED users. The results also describe this inaccurately with respect to figure 1 if this is truly a percent.”*

We thank the reviewer to highlight this point. Percentages have been calculated using the ratio: amount of older ED users with a ER2 score/ total amount of older adults who visited ED. We apologize for any confusion that have been caused by the formulation. Please see changes on page 10.

**Comment 4:***“Similarly, on page 9, hospital admission rate is described as proportion of patients admitted to the hospital divided by the proportion discharged to the ED. The denominator should be all patients. I also don’t know that discharged to the ED makes sense. I think it would be discharged to home or anywhere other than the hospital, but this is irrelevant since the denominator for a rate should be all patients.”*

As per comment 3, the reviewer is right. The ratio used to calculate the hospital admission rate was: amount of older ED users admitted to hospital/total amount of older adults who have visited ED. Please see changes on page 10 and see also answer to the comment 3.

**Comment 5:** *“I’m now more confused about what happened to people who died during their hospitalization. The description of the sample indicates that death during hospitalization was an exclusion criterion. The description of Kaplan-Meier curves now says it’s a censoring criterion. Which is correct? An outcome like hospital-free days over a 2-4 weeks period could have advantages in that it would capture death as a severe outcome (no hospital free days), but I don’t feel strongly about this. The discrepancy just needs to be addressed. I can understand that individuals who died would need to be excluded from certain analyses. Considering them to have a short length of stay due to rapid death would be a problem for the LOS analyses.”*

The reviewer underscored an important point. Older patient that died during their hospitalization (i.e., in ER or on the wards) have not been included in this study. Indeed, our main purpose was to examine if ER2 was usable in ER and the association with long LOS. As a matter of fact, patient that died could not represent the studied population. We did not include the patients that died in our analyses. Please see changes on page 12.

**Comment 6:** *“The p-values shown in figure 1 do not seem to be accurately reflected in the results. October vs. November is described as significant in the results, but the p-value provided is for October vs. December. This also appears significant. All told, figure 1 seems to suggest that completion rates were highest in October then declined slightly in subsequent months. The conclusion that completion rates increased is basically accurate when considering the lower rate of completion in the first month of implementation, but the numbers really seem to reflect an increase in month 2 followed by a decrease in month 3, with a non-significant trend toward another increase in month 4 that still lags behind month 2.”*

We thanks the reviewer for this comment. There is a significant decrease (P≤0.001) of the percentages of completed ER^2^ assessment between October and November but it was an exception. It is important to consider: 1) September as the referent month (there was a significant increase of the ER2 completion when comparing to this month), 2) the global trend of the filling rate of this tool during the 4-month period of the study which is a significant improvement. This finding suggests that there is a learning curve and that ED nurses may require a training period to implement ER^2^ in their daily practice. Please see corrections on page 12

**Comment 7:** *“For the models in table 2, what is the purpose of including model 1, which is actually three different models, instead of just using model 2? Why would you merge low and high risk together as a reference group and compare the moderate risk group? That is intermediate vs. the other two groups, so the comparison makes little sense. I understand the sample size of the moderate risk group may be a concern that could lead with lumping with low or high risk, but model 2 uses a much more typical approach that is interpretable to readers, while model(s) 1 uses an unusual and confusing approach that adds little useful information in my opinion. I can’t think of any reason to include model(s) 1. If you’re interested in contrasts of high vs. medium risk, these could be examined as contrasts within model 2. The sample size for comparisons of the low risk vs. high risk group are more than adequate without lumping them with the medium risk group.”*

As per comment 1, we removed the model 1 in table 2 to make the results clearer. The purpose of the Model 1 was to describe, without adjustment, the association of the length of stay and in hospital as well as hospital admission with the different risk levels of ER2. We agree that the main information is provided by the Model 2 and the comparison between the low and the moderate/high risk levels. Please see changes on page 19 and see also answer to the comment 1.

**Comment 8:** *“In table 2 footnotes, what does it mean that this was “Separated model for each Emergency Room Evaluation and Recommendation risk level”? Are you saying the model was stratified by this variable? If so this should be described in the methods. It does raise some questions about why adjustment was inadequate and whether there was effect modification by risk level, but overall I think a stratified model is reasonable.”*

We apologize for this misunderstanding. We indicated that the difference between these two models was based on the referent group. The analyses in the Model 1 have been done without a referent level and used separated level of risk whereas the Model 2 used low risk level as the referent level.

**Comment 9:***“The abstract is confusing because of the way the results are presented. The lengths of stay in hours look like actual lengths of stay instead of the difference in length of stay. Please rewrite this for clarity.”*

We agree with the reviewer and made the changes. Low-risk group was associated with a short LOS in ED (approximately 3.7 less hours; P≤0.001) and in hospital (approximately 4.5 less days; P=0.002), whereas the high-risk group was associated with a long LOS in ED (approximately 3.6 more hours; P≤0.001) and in hospital (approximately 4.3 more days; P<0.003) compared to the other risk groups of patients. Please see changes on page 3.

**Comment 10:** *“*I don’t understand the meaning of “hospital health areas.” This needs to be described somewhere.”

We apologize for this lack of information. “Hospital health areas” is specific to Quebec’s healthcare system and refers to the hospital they are assigned to, according to where they live. However, they can visit ED from other health areas than their own. Please see changes on pages 8, 9, 11 and 18.

**Comment 11:** *“There are occasional issues with grammar and usage in the paper. Careful review of the paper by someone with English as a primary language would be helpful, though many of these issues could be addressed at the text editing stage.*”

A revision of the manuscript has been made by a native English physician. Please see corrections through the revised manuscript.
