# Supplementary material for: Risk for short-term undesirable outcomes in older emergency department users: Results of the ER2 observational cohort study
Source: PLoS One. 2021 Aug 11;16(8):e0249882. doi: 10.1371/journal.pone.0249882 (PMC8357090; doi:10.1371/journal.pone.0249882)
Supplement: Attachment — Submitted filename: Responses_Comments_PONE-D-20-15983-R4.docx [file pone.0249882.s003.docx]

Manuscript:

- ID#: **PONE-D-20-15983**
- MS TITLE: **"Risk for short-term undesirable outcomes in older emergency department users: Results of the ER2 observational cohort study"**
- Submitted for publication in **PLOS ONE**

We thank the Reviewer for her/his second review. As suggested, we performed a revision of our manuscript. Please, find below our point-by-point responses to the comments and queries raised by the Reviewer.

**Comment of the Reviewer**

**Comment 1:** *“The manuscript presentation is improved…” but there are still a few issues that need attention.”*

We thank the reviewer for this positive comment on our manuscript.

**Comment 2:** *“…but there are still a few issues that need attention.”*

We made requested changes as suggested in Reviewer’s comments below. Please see corrections through the revised manuscript.

**Comment 3:** *“The results in the abstract appear to reflect the old models that have been removed from the paper, as they use both other groups as the reference. Please update these to reflect the results in the new table 2 (medium vs. low and high vs. low).”*

The reviewer is correct and we apologize for those mistakes. We made the requested changes. Please see corrections on pages 3 and 4.

**Comment 4:** *“I don’t see strong support for the following sentence.
“It suggests that older ED users who will stay a shorter period of time in ED or in hospital have a phenotype of middle-age patients with few and less severe acute diseases and minimal to no disability.”. I get the idea that the lower risk patients were possibly less likely to be old-old but calling them middle-age makes one think of a middle-aged person. I don’t think any patients in this study would be classified as middle-aged per the usual definition. The age difference also isn’t that large. A mean age of 83 in the low-risk group doesn’t seem like a middle age vs. others. I don’t see categorical age in table 1, so it’s hard to phenotype patients in terms of their likelihood to be a particular age. They’re generally slightly younger, but that’s about all that can be concluded”*

We apologize for this misunderstanding. The reviewer is correct, the population that has been included in this study can not be called middle-age. We wanted to point out that patients with a shorter period of time in ED were not oldest old patients. We think that middle-old patients is more appropriate to characterize this age group. Please see changes on pages 22-23.

**Comment 5:***“* *Similarly, I don’t think data are presented to support the idea that they have minimal to no disability. Only use of walking aids and use of home support are included in the tool, and the latter is a minor criterion that could leave someone in the low-risk group. There are many kinds of disability beyond need to use walking aids.”*

We agree with the reviewer and we removed this sentence. Please see changes on page 22.

**Comment 6:** *“Also, what data supports that they have less severe acute diseases? This is perhaps reasonable to assume because of the lower likelihood of hospitalization, but it doesn’t seem to have much support from table 1. There are only limited differences in the triage scale and they sometimes suggest low scoring patients were more acutely ill in some respects, even if they were less frail. The reasons for the ED visit don’t vary that much. It could be that frailty matters as much or more than the conditions themselves, at least as they’re classified in the data presented.”*

The reviewer underscored an important point. It is highly probable that the patient's level of frailty was associated with the severity of acute diseases. Indeed, it has been shown that the level of frailty is associated with more severe acute conditions, a higher risk of short-term adverse events and a higher risk of death. Please see changes on pages 22-23.

**Comment 7:** *“Page 18. It's a little awkward to write in this sentence that the low-risk group was the reference group. That's noted in the methods. “In addition, regarding prolonged LOS, using the low-risk group as reference showed that the high-risk group was associated with a longer LOS in ED… I think this would read better if it just said LOS in the ED and hospital was longer in the high-risk compared to the low-risk group.”.”*

We thank the reviewer for this comment. We followed his recommendations and we have modified the sentence to clarify. Please see corrections on page 18.

**Comment 8:** *“I'm not sure if the statement that one can email the author with proper approvals to get the data is adequate to meet the data sharing policy requirements.  Not including the original data with ages can be understood since some of them are identifiable (90 or older). Suggest the key data elements used in the manuscript could be supplied deidentified with categorical ages if public sharing is crucial, e.g. 65-74, 75-84, 85+..”*

We thank the reviewer for the opportunity to clarify this point. If a researcher wants to access the database, a formal protocol needs to be sent to the databank owner. A scientific evaluation will be done and in case of agreement, the researcher will have to communicate an ethical agreement for the protocol. The databank owner will then proceed to the signature of a Data Transfer Agreement with the researcher.
Data will be sent only under these 3 conditions: 1/ scientific approval from the databank owner, 2/ approval form the applicant’ s ethic committee and 3/ data transfer agreement duly signed by both parts. Age will be identified in shared databases by using categorical ages (65-74, 75-84, 85+).
